# Effect of hydrogen/oxygen therapy for ordinary COVID-19 patients: a propensity-score matched case-control study

**DOI:** 10.1186/s12879-023-08424-4

**Published:** 2023-06-29

**Authors:** Yingying Zeng, Weijie Guan, Kai Wang, Zhijun Jie, Xu Zou, Xiaoping Tan, Xinyu Li, Xiaohua Chen, Xiaoting Ren, Junhong Jiang, Zeguang Zheng, Jindong Shi, Nanshan Zhong

**Affiliations:** 1 Department of Respiratory and Critical Care Medicine, Fifth People’s Hospital of Shanghai, Fudan University, 801 Heqing Road, Shanghai, 200240 China; 2 Center of Community-Based Health Research, Fudan University, Shanghai, China; 3 State Key Laboratory of Respiratory Disease, National Clinical Research Center for Respiratory Disease, Guangzhou Institute of Respiratory Health, First Affiliated Hospital of Guangzhou Medical University, 151 Yanjiang Road, Guangzhou, 510000 China; 4 Department of Critical Care Medicine, Guangdong Province Hospital of Traditional Chinese Medicine, Guangzhou, China; 5 Department of Respiratory and Critical Care Medicine, Jiangling County People’s Hospital, Jingzhou, Hubei China; 6 Department of Infectious Diseases, Shanghai Pudong New Area Gongli Hospital, Shanghai, China; 7 Department of Infectious Diseases, Shanghai Jiaotong University Affiliated Sixth People Hospital, Shanghai, China; 8 Department of Respiratory and Critical Care Medicine, Dushu Lake Hospital Affiliated to Soochow University, Jiangsu, China

**Keywords:** Hydrogen/oxygen therapy, Ordinary COVID-19 patients, Propensity-score matched, Length of hospitalization

## Abstract

**Background:**

Hydrogen/oxygen therapy contribute to ameliorate dyspnea and disease progression in patients with respiratory diseases. Therefore, we hypothesized that hydrogen/oxygen therapy for ordinary coronavirus disease 2019 (COVID-19) patients might reduce the length of hospitalization and increase hospital discharge rates.

**Methods:**

This retrospective, propensity-score matched (PSM) case–control study included 180 patients hospitalized with COVID-19 from 3 centers. After assigned in 1:2 ratios by PSM, 33 patients received hydrogen/oxygen therapy and 55 patients received oxygen therapy included in this study. Primary endpoint was the length of hospitalization. Secondary endpoints were hospital discharge rates and oxygen saturation (SpO_2_). Vital signs and respiratory symptoms were also observed.

**Results:**

Findings confirmed a significantly lower median length of hospitalization (HR = 1.91; 95% CIs, 1.25–2.92; *p* < 0.05) in the hydrogen/oxygen group (12 days; 95% CI, 9–15) versus the oxygen group (13 days; 95% CI, 11–20). The higher hospital discharge rates were observed in the hydrogen/oxygen group at 21 days (93.9% vs. 74.5%; *p* < 0.05) and 28 days (97.0% vs. 85.5%; *p* < 0.05) compared with the oxygen group, except for 14 days (69.7% vs. 56.4%). After 5-day therapy, patients in hydrogen/oxygen group exhibited a higher level of SpO_2_ compared with that in the oxygen group (98.5%±0.56% vs. 97.8%±1.0%; *p* < 0.001). In subgroup analysis of patients received hydrogen/oxygen, patients aged < 55 years (*p* = 0.028) and without comorbidities (*p* = 0.002) exhibited a shorter hospitalization (median 10 days).

**Conclusion:**

This study indicated that hydrogen/oxygen might be a useful therapeutic medical gas to enhance SpO_2_ and shorten length of hospitalization in patients with ordinary COVID-19. Younger patients or those without comorbidities are likely to benefit more from hydrogen/oxygen therapy.

**Supplementary Information:**

The online version contains supplementary material available at 10.1186/s12879-023-08424-4.

## Background

Coronavirus disease 2019 (COVID-19) pandemic is caused by severe acute respiratory syndrome coronavirus 2 (SARS-CoV-2) and characterized by an interstitial pneumonia and altered vascular permeability that may lead to a severe and even fatal outcome [1, 2]. Within months, it has caused a global pandemic and posed a major threat to public health worldwide [3, 4]. The clinical manifestations of COVID-19 are diverse, the typical clinical features of COVID-19 mainly include fever (not in all), coughing, dyspnea, and myalgia [5–7]. Terribly, patients with dyspnea and hypoxemia can rapidly deteriorate into acute respiratory distress syndrome, respiratory failure, severe sepsis with septic shock, and even multiple organ failure, only within 1 week [4, 8, 9]. Despite several agents (e.g., nirmatrelvir–ritonavir) has been authorized for emergency use for the treatment of COVID-19, the supply falls short of the global demand [10]. More crucially, current therapies provide limited clinical benefits so far [11], which creates a need for more and novel options.

Therapeutic medical gas as pharmaceutical gaseous molecules are emerging as a novel and innovative therapeutic tool [12]. Oxygen as a therapeutic agent has been recommended to the treatment of respiratory distress, hypoxemia, or shock [13]. Oxygen therapy remains one main adjuvant therapy for COVID-19, particularly for hypoxaemia, until suitable anti-infective therapies become available [14]. However, oxygen therapy might cause the accumulation of distal bronchial viscous secretions because of positive pressure ventilation mode, and thus increase airway resistance, aggravate systemic hypoxia [15]. In recent years, molecular hydrogen treatment has the potential to preventive and therapeutic applications against many diseases due to its extensive effects, such as antioxidant, anti-inflammatory, anti-apoptotic [16, 17]. More importantly, the small molecular properties of hydrogen enable it can rapidly reaches the alveoli, suggest a unique advantage for lung disease [18].

Encouragingly, a review of the research suggest that the early inhalation of hydrogen may mitigate lung injury, promote viscous sputum drainage and reduce airway resistance [19]. While, Xu et al., reported that an important mechanism contributing to dyspnea and disease progression in patients with COVID-19 might be the heightened airway resistance leads to increased work of breathing [20]. Therefore, Hydrogen therapy has the potential to ameliorate the respiratory symptoms and prevent against the disease progression, which become a new adjuvant therapy for COVID-19. However, the level of evidence for hydrogen therapy for COVID-19 is inadequate. There, so far, only an open-label multicenter clinical trial first verified that Hydrogen/oxygen inhalation can relief dyspnea and other respiratory symptoms in patients with COVID-19, regardless of the disease severity. Nonetheless, the design of the study was limited because of neither randomly assigned dyspnea patients with COVID-19 nor matched the patients with propensity scores, which could have resulted in selection bias [21]. What is noteworthy is that approximately 60–90% of hospitalized patients with COVID-19 have comorbidities [22–24], which cause the length of hospitalization and mortality increased, and thus the costs for hospitalization increased concurrently. Moreover, a large number of epidemiological investigations indicated that the largest proportion of patients with COVID-19 are ordinary and moderate [25], thus it is of great significance to treat this population to help control epidemic progression.

Therefore, this study aimed to compare the efficacy of hydrogen/oxygen therapy and oxygen therapy in patients with ordinary COVID-19. Additionally, a subgroup analysis was performed to identify the potential population that are likely to benefit most from this therapy.

## Method

### Study design

This retrospective, propensity-score matched (PSM) case-control study assessed the impact of Hydrogen/oxygen therapy on the length of hospitalization in patients with ordinary COVID-19. Total 180 patients were collected from 3 centers in China, including 42 patients received hydrogen/oxygen therapy and 138 patients received oxygen therapy on the basis of standard-of-care. The study has been performed in accordance with the Declaration of Helsinki. The protocol was approved by the Ethics Review Committee of Shanghai Fifth People’s Hospital affiliated to Fudan University (Ethical review: 2020 − 132). The study was exempted informed consent by the Ethics Review Committee of Shanghai Fifth People’s Hospital affiliated to Fudan University due to the retrospective nature of the study.

### Subject eligibility

Ordinary COVID-19 patients were collected for this study with aged 18 years or older. Ordinary-type patients of COVID-19 were diagnostic refer to the Diagnosis and Treatment Program of New Coronary Pneumonia (the sixth edition) published by the National Health Commission of China [20], that is defined as mild clinical symptoms: fever, cough, sore throat, mild fatigue, impairment of smell and taste, while having typical ground-glass opacities but no signs of severe pneumonia. Patients were eligible for enrollment in the hydrogen/oxygen therapy group if they inhaled hydrogen/oxygen (H_2_-O_2_) within 3 days of admission. In addition, patients who inhaled H_2_-O_2_ for at least 7 days during hospitalization was also eligible for this study. During the same study period, patients were included in oxygen therapy group if patients received oxygen (O_2_) therapy within 3 days of admission and received oxygen (O_2_) for at least 7 days during hospitalization.

### Intervention

All eligible patients were assigned in 1:2 ratios by PSM analyses. After matching was established, patients were assigned to hydrogen/oxygen group (n = 33) and oxygen group (n = 55). All eligible patients received the standard-of-care according to the Diagnosis and Treatment Program of New Coronary Pneumonia (the sixth edition) [20]. On the basis of standard-of-care, patients in hydrogen/oxygen group inhaled hydrogen/oxygen (hydrogen/oxygen volume ratio of 2:1) using a Hydrogen/Oxygen Generator (model AMS-H-03, Shanghai Asclepius Meditec Co., Ltd., China). Hydrogen/oxygen was administered at flow rate of 3.0 L/min for more than 7 consecutive days and a minimum of 6 h/day. Patients in the oxygen group received oxygen therapy (air/oxygen volume ratio of 2:1) at low flow rate of < 3.0 L/min. The treatment period of oxygen therapy is adjusted according to the clinical situation. Both hydrogen/oxygen mixture and oxygen were introduced via a nasal mask.

### Outcomes and assessment

The primary efficacy endpoint was the length of hospitalization. The discharge criteria for COVID-19 are based on Diagnosis and treatment of COVID-19 in China [20]. The criteria for hospital discharge were as following: the blood oxygen saturation and body temperature has returned to normal levels, and negative polymerase chain reaction (PCR) tests on two consecutive occasions as well as the lesion absorbed essentially.

Secondary endpoint was hospital discharge rates at 14, 21, and 28 days, and oxygen saturation (SpO_2_) on the day 5 of admission. Additionally, the vital signs (systolic and diastolic blood pressure [BP], temperature, heart rate and respiratory rate) and respiratory symptoms (cough and shortness of breath) were also investigated. In addition, we retrospectively collected data on adverse events, vital signs and deaths during hospitalization.

### Statistical analysis

PSM was used to control for the baseline confounders between hydrogen/oxygen therapy group and oxygen therapy group. The propensity score (PS) was calculated using a logistic regression model with variables including gender, age, and the days from onset to hospitalization. A 1:2 ratios match (hydrogen/oxygen group to oxygen group) was performed on the estimated PS using Greedy matching method with a caliper width of 0.2 on the PS scale. Covariate balance was assessed with standardized differences, with meaningful imbalances set at values higher than 10%.

According to the normality of data distribution, nominal variables were presented as n (% frequency of group) and continuous variables as means ± SD or medians (interquartile range). Analysis of nominal variables were performed using the chi-square test or Fisher exact test for between-group comparisons. Comparison of continuous variables between two groups were analyzed using t-test. Length of hospitalization curves were prepared using the Kaplan-Meier method, and a comparison of the length of hospitalization between hydrogen/oxygen therapy group and oxygen therapy group was made using the log-rank test. Hazard ratios (HR) and associated 95% confidence intervals (CIs) were assessed with the use of a stratified Cox proportional-hazards model. Sensitivity analyses also performed for primary endpoint in the unmatched patients from real-world setting and 1:1 matched patients. Additionally, in all patients who received hydrogen/oxygen therapy, the stratified log-rank test was used to access differences in the length of hospitalization among age, gender, days from onset to hospitalization, and comorbidities. All statistical tests are two-sided, *p* value of < 0.05 was considered statistically significant. All statistical analyses were conducted using SAS software version 9.4 (SAS Institute Inc, USA).

## Results

### Patients

**Fig. 1:**
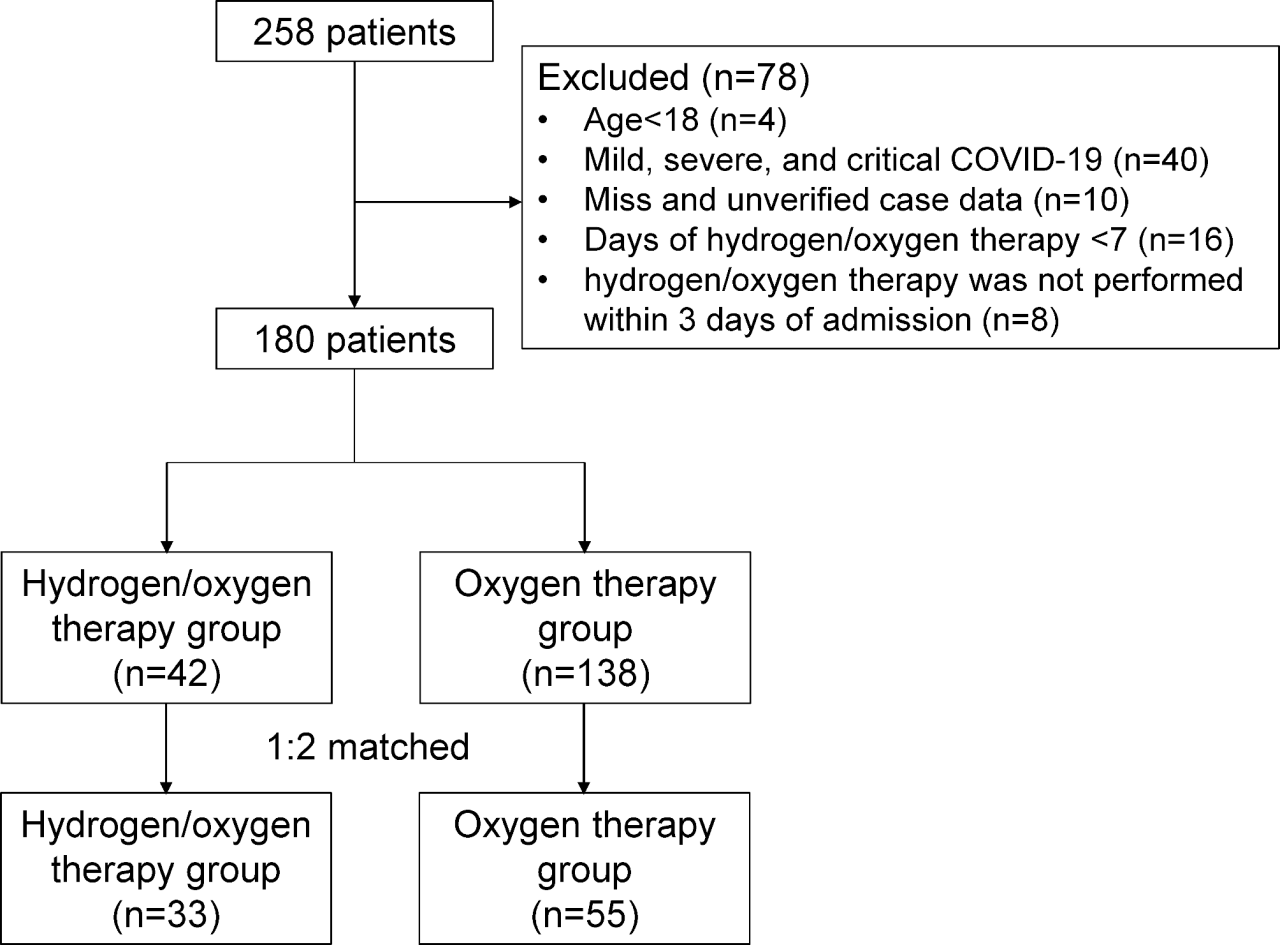
Study profile

Between December 28, 2019 and March 24, 2020, the initial study included 258 patients hospitalized with COVID-19 from 3 centers. After data cleaning, 180 patients were included in this study. Study profile was shown in Fig. 1. Among them, 42 patients were given hydrogen/oxygen therapy, 138 patients were oxygen therapy. The comparison of characteristics before PS match and after 1:1 PS match between hydrogen/oxygen group and oxygen group was shown in Additional file 1: Table S1.

PSM of the eligible patients at 1:2 ratio, using gender, age, and the days from onset to hospitalization as variables, yielded 33 and 55 patients in the hydrogen /oxygen therapy group and oxygen therapy group, respectively. After propensity score matching, the groups were comparable in terms of the baseline characteristics (Table 1). There was no significant difference among clinical symptoms, antiviral treatment, vital signs, and routine laboratory examination on admission between two groups (*p* > 0.05).

**Table 1:** Baseline clinical characteristics of patients after propensity score matching

Variables	Hydrogen/oxygen group (n = 33)	Control group (n = 55)	*P* value
Age	54.7 (9.9)	53.0 (13.7)	0.527
Gender (male/female)-n (%)	12 (36.4)/21 (63.6)	25 (45.5)/30 (54.5)	0.403
Comorbidities (n)	0.8 (1.1)	0.9 (1.2)	0.736
Days from onset to hospitalization (d)	23.6 (12.4)	23.5 (13.4)	0.884
Clinical symptoms, yes-n (%)			
Fever	2 (6.1)	8 (14.5)	0.309
Respiratory symptoms	22 (66.7)	38 (69.1)	0.813
other symptoms	18 (54.5)	37 (67.3)	0.233
Vital signs			
Respiratory rate (bpm)	20 (2.0)	20 (1.0)	0.313
Heart rate (rpm)	86.4 (13.1)	84.2 (14.5)	0.470
Systolic pressure (mmHg)	134 (17.0)	131 (15.0)	0.392
Diastolic pressure (mmHg)	84 (9.0)	82 (11.0)	0.270
Temperature (℃)	36.6 (0.4)	36.7 (0.6)	0.322
Laboratory examination			
WBC (×10^9^/L)	5.4 (1.4)	5.3 (1.8)	0.784
Lymphocyte count (×10^9^/L)	1.6 (0.4)	3.7 (15.1)	0.744
RBC (×10^12^/L)	4.2 (0.7)	4.2 (0.7)	0.808
Hb (g/L)	130 (16.0)	126 (19.0)	0.989
PLT (×10^9^/L)	223 (70.0)	239 (83.0)	0.379
CRP (mg/L)	1.7 (2.9)	1.9 (4.8)	0.318
ALT (U/L)	36 (29.0)	31(30.0)	0.339
AST (U/L)	26 (13.0)	25 (15.0)	0.478
BUN (mmol/L)	4.4 (1.3)	4.7 (1.6)	0.381
Scr (µmol/L)	61.1 (16.0)	64.5 (15.1)	0.322
LDH (U/L)	190 (48.0)	195 (59.0)	0.733
Antiviral therapy-n (%)	9 (27.3)	25 (45.5)	0.090
Oxygen saturation (%)	98.1 (0.9)	97.4 (1.6)	0.056

Data were expressed as mean (standard deviation) or n (%). WBC, blood cell count; RBC, red blood cell count; Hb, hemoglobin; CRP, C-reactive protein; ALT, alanine aminotransferase; AST, aspartate aminotransferase; BUN, blood urea nitrogen; Scr, serum creatinine; LDH, lactate dehydrogenase

### Clinical outcomes and safety analysis

**Fig. 2:**
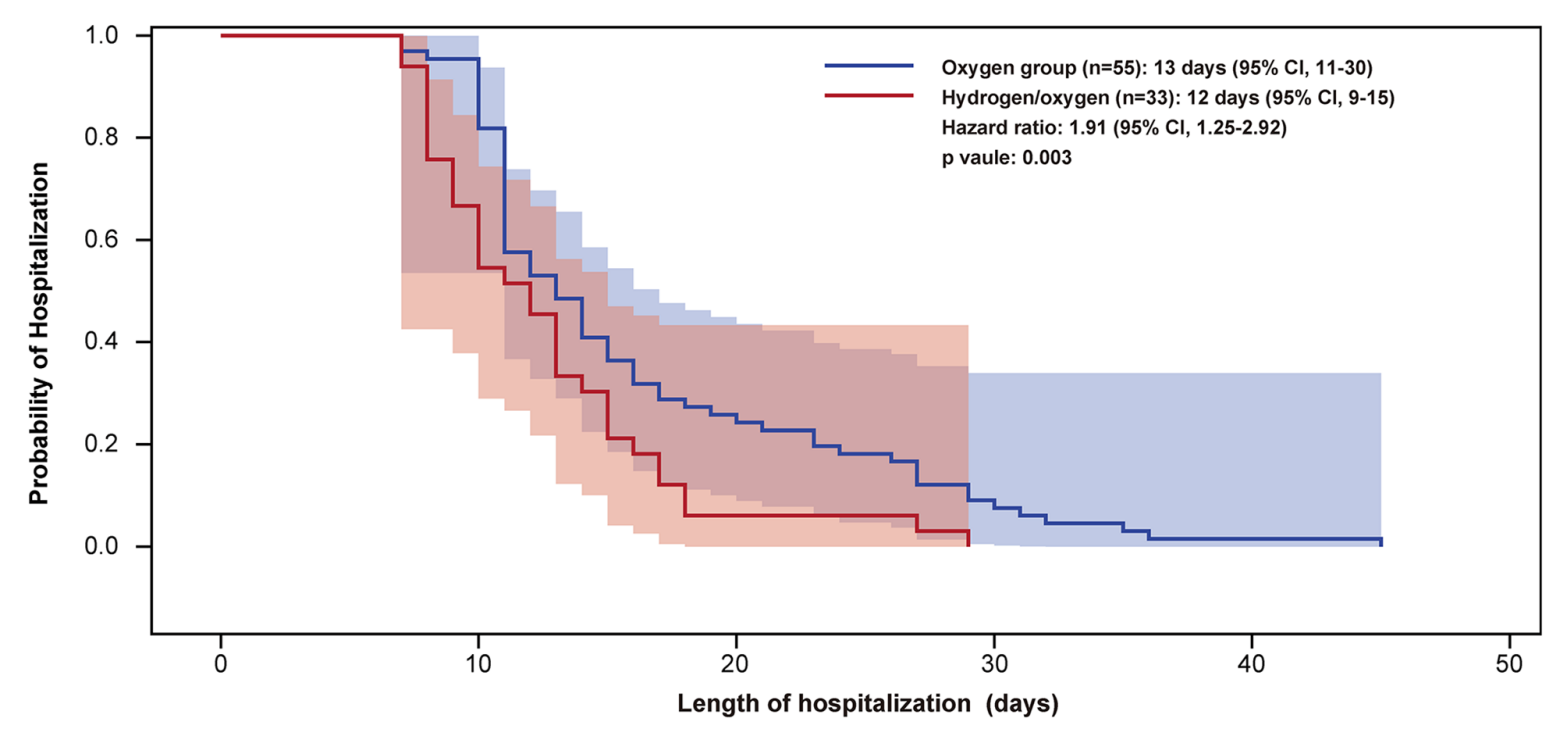
Kaplan-Meier curves of the length of hospitalization in ordinary COVID-19 patients (1:2 matched)

In the entire matched group, the median length of hospitalization for patients who inhaled hydrogen/oxygen were 12 days (95% CI, 9–15) and patients who inhaled oxygen were 13 days (95% CI, 11–20), with a HR of 1.91 (95% CI, 1.25–2.92; *p* < 0.05; Fig. 2). The length of hospitalization in unmatched patients from real-world setting and 1:1 matched patients were also analyzed as sensitivity analysis. The 4.5-day shorten with hydrogen/oxygen therapy was observed in the real-world data from all unmatched patients (11.5 days [95%CI: 10–13] vs. 16 days [95%CI: 14–18]; *p* < 0.001; Additional file 1: Fig. S1A) and 2-day shorten in the 1:1 matched patients (12 days [95%CI: 9–14] vs. 14 days [95%CI: 11–16]; *p* = 0.019; Additional file 1: Fig. S1B).

With regard to the hospital discharge rates (Table 2), there was no significant difference at 14 days between two groups (hydrogen/oxygen = 69.7% vs. oxygen = 56.4%) with a HR of 1.66 (95% CI, 0.96–2.88; *p* = 0.072). However, patients received hydrogen/oxygen therapy resulted in significantly higher hospital discharge rates at 21 days (93.9% vs. 74.5%; HR = 1.92; 95% CI, 0.21–3.04; *p* = 0.005) and 28 days (97.0% vs. 85.5%; HR = 1.85; 95% CI, 1.17–2.92; *p* = 0.008), compared with patients received oxygen.

**Table 2:** The hospital discharge rates of hydrogen/oxygen therapy and oxygen therapy

Discharge time (days)	Hydrogen/oxygen therapy No. (%)	Oxygen therapy No. (%)	Hazard ratios (95%CI)	*P* value
14	23 (69.7)	31 (56.4)	1.66 (0.96, 2.88)	0.072
21	31 (93.9)	41 (74.5)	1.92 (1.21, 3.04)	0.005
28	32 (97.0)	47 (85.5)	1.85 (1.17, 2.92)	0.008

Regarding the laboratory data, after the 5-day treatment, SpO_2_ were found to be significantly higher in the hydrogen/oxygen group (98.5%±0.56%) than that in oxygen group (97.8%±1.0%), with a significant difference (*p* < 0.001). Only 1 patient in each group (hydrogen/oxygen = 3.0% vs. oxygen = 1.8%) has developed into severe disease with respiratory failure (*p* > 0.999). No death and significant adverse effects were observed in both groups. There results indicated that the hydrogen/oxygen therapy would not increase the risk of AEs in the COVID-19 treatment.

### Subgroup analysis

Considering that patients with different age, gender, days between onset and hospitalization, and complications might have different effect on the length of hospitalization to hydrogen/oxygen therapy, we performed the subgroup analyses in all patients who were given hydrogen/oxygen therapy (Table 3). The subgroup results showed that patients aged ≥ 55 years links to significantly longer hospitalization compared with patients aged < 55 years (14 days [95% CI, 7–17] vs. 10 days [95% CI, 9–13]; *p* = 0.028). Additionally, patients without comorbidities exhibited a shorter hospitalization than those with comorbidities (10 days [95% CI, 9–12] vs. 15.5 days [95% CI, 10–17]; *p* = 0.002). However, the gender (*p* = 0.684) and the days from onset to hospitalization (*p* = 0.140) were not identified to be associated with the length of hospitalization after hydrogen/oxygen therapy.

**Table 3:** Outcomes of subgroup analyses of hydrogen/oxygen therapy in ordinary COVID-19 patients

Group	Median length of hospitalization (95% CI)	*P* value
Age		0.028
≥ 55 (n = 21)	14.0 (9.0, 17.0)	
< 55 (n = 21)	10.0 (9.0, 13.0)	
Gender		0.684
Male (n = 14)	13.0 (8.0, 16.0)	
Female (n = 28)	10.0 (10.0, 13.0)	
Comorbidities		0.002
With comorbidities (n = 18)	15.5 (10.0, 17.0)	
Without comorbidities (n = 24)	10.0 (9.0, 12.0)	
Duration between onset and hospitalization		0.140
0–14 days (n = 8)	12.5 (9.0, 27.0)	
> 14 days (n = 34)	10.0 (9.0, 14.0)	

## Discussion

To our knowledge, this is the first retrospective, multicenter, PMS case-control study investigated the impact of hydrogen/oxygen therapy on length of hospitalization in ordinary COVID-19 patients. The study revealed that hydrogen/oxygen inhalation might reduce length of hospitalization and increase hospital discharge rates of ordinary COVID-19 patients.

The pandemic of COVID-19 has placed an unprecedented strain on health authorities across the world. The rapid spread of the SARS-CoV-2 virus throughout the world caused the COVID-19 patients needing hospitalization have increase rapidly [26]. The fear, however, is that an increased length of hospitalization poses a major challenge to the health system. On the one hand, it prolonged COVID-19 exposure for patients and health care providers, and thus increases the rate of infection [27]. On the other hand, an increased duration of hospital stay reduce available beds available beds for other patients, increases use of facemasks and protective clothing, and reduce the effective allocation rate of resources, which caused the healthcare services being overwhelmed [28]. Additionally, a longer length of hospitalization means a greater medical costs, and thus lead to patients being overwhelmed. Therefore, shorten the length of hospitalization of COVID-19 patients may improve resource utilization, reduce the rate of infection and ultimately reduce pressure on health systems. Excitingly, the most striking finding of the present study is that hydrogen/oxygen therapy can shorten the length of hospitalization. Although only 1-day shorten in median length of hospitalization is not striking in primary analysis, the results of sensitivity analysis remain support the efficacy of hydrogen/oxygen therapy with more notable improvement, particularly in real-world setting (4.5 days). From the perspective of data distribution, we found that the median length of hospitalization in hydrogen/oxygen group had a narrower 95% CI and smaller upper/lower margin compared to that in the oxygen group (95% CI, 9–15 days vs. 95% CI, 11–20 days), suggesting more patients in the hydrogen/oxygen group had relatively shorter length of hospitalization. The higher hospital discharge rates at 21 days (93.9% vs. 74.5%; HR = 1.92; p = 0.005) also supported this hypothesis. Overall, hydrogen/oxygen has superiority than oxygen in shortening length of hospitalization for patient with ordinary COVID-19.

As revealed by a review report, up to 75% of patients with COVID-19 require oxygen support due to respiratory dysfunction [29, 30]. Moreover, nocturnal oxygen therapy can inhibit the rapid replication of the virus and improve the body’s antiviral ability at early COVID-19 [31]. However, many asymptomatic or mild COVID-19 patients were rapidly developed into respiratory failure and acute respiratory distress only within 1 weeks [32]. While oxygen therapy cannot be rapidly ameliorated symptoms and prevent against the disease progression. We speculated that the superiority of hydrogen in rapid ameliorated symptoms of COVID-19 and prevent against the disease progression primarily because its low density could reduce the resistance to flow in the airways, improve airway resistance, and thus relieve dyspnea and increase the SpO_2_ [33]. The improvement in SpO_2_ were also observed in our study, but we only recorded the SpO_2_ after 5-day treatment due to the emergency. Despite the result is preliminary and not robust, the previous studies also demonstrated the improvement in SpO2 after hydrogen inhalation [21, 34], providing additional evidence for this finding. Furthermore, the property of small molecules enable hydrogen to diffuse rapidly to get places most other antioxidants cannot [35], which may contribute to the fast-acting of therapeutic effect and reduce length of hospitalization. Previously study confirmed that inhale hydrogen/oxygen can significantly relief dyspnea and other respiratory symptoms of COVID-19 patients as early as days 2 and 3 [36]. This also supported our findings. Meanwhile, the anti-inflammatory, anti-apoptotic and anti-oxidative functions of hydrogen can protect multiple organs including the kidney, heart, and nervous system, and thus maintain the normal response of the body, reduce mortality and increase hospital discharge rates [19]. Nevertheless, this explanation cannot be verified from present study because we did not collect the data of inflammatory and oxidative factors during treatment period. This study further verified the efficacy of hydrogen/oxygen therapy in patients with ordinary COVID-19 according to the length of hospitalization and discharge rate of patients. Presently, several studies have focused on the treatment of moderate or severe COVID-19 patients, while less studies have investigated on ordinary COVID-19 patients [36–40]. Therefore, the study also expanded the adaptive patient population of COVID-19 who received hydrogen/oxygen therapy.

In addition, subgroup analyses in hydrogen/oxygen therapy group further verified that older age (> 55 years) and a high number of comorbidities were associated with the length of hospitalization, but not associated with gender and days between onset and hospitalization. Several previously studies have also found that older age and comorbidities appeared to be associated with prolonged hospitalization [25, 35, 41–43], which was consistent with our study. We speculated that older ages and a high number of comorbidities might leads to the ability of antiviral immune response decreased and thus exhibit negative effect on hydrogen/oxygen therapy. Prior study also suggested that comorbidities such as hypertension, diabetes and heart diseases might impair the functions of immune cells, thus leading to suppressed immunological function [44]. Moreover, older age appeared to be associated with the increased risk of complication, the likely reasons belong to older age less robust immune responses and more susceptible to disease [45]. Accordingly, we recommend surveillance of COVID-19 patients with comorbidities and older ages during hydrogen/oxygen therapy. However, the data were only from this small number of patients; thereby, further investigations for impact of the ages and comorbidities to hydrogen/oxygen therapy are imperative. On the contrast, the therapeutic effects of hydrogen/oxygen was not associated with gender and days between onset and hospitalization. A retrospective cross-sectional study also supported this findings [46]. It highlighted that the general applicability of hydrogen/oxygen therapy in the majority of patients with ordinary COVID-19. Although gender and days between onset and hospitalization were not associated with length of hospitalization, more evidence and randomized trials are needed to draw definitive conclusions. Furthermore, there no significant adverse effects were observed in hydrogen/oxygen therapy, and the hydrogen/oxygen therapy regimens present acceptable safety and tolerability profile. Thus, we could conclude that hydrogen/oxygen therapy has the potential to be a novel and effective treatment for COVID-19.

Although this present study collect dataset from 3 center to balance the selection bias by PS matching method, which makes our research more robust. But there still exist limitations. Firstly, this was a retrospective study and lack of randomization. Although we attempted to mitigate bias by performing PS matching on key baseline characteristics, we cannot exclude the influence of unmeasured confounding factors on the study outcomes. Secondly, interpretation of our findings might be limited by the sample size the patients of the study included was relatively low. Besides, due to the emergency at the earliest stage of the epidemic outbreak, the unavailability of post-treatment laboratory data as well as the incompleteness of SpO_2_ data were another limitation of this study, which led to the lack of sufficient evidence and weakened the strength of conclusion. Thus, our conclusion should be interpreted with caution and require confirmation by larger studies.

## Conclusions

In summary, this PSM case–control study demonstrated the superiority of hydrogen/oxygen inhalation over oxygen in shortening length of hospitalization for patient with ordinary COVID-19, particularly for younger patients or those without comorbidities. This finding suggested that hydrogen/oxygen therapy might be considered as a therapeutic option, and have the potential to reduce pressure on medical resources and health systems in the management of COVID-19. Nevertheless, due to the limited sample size and inherent limitations of retrospective study, further studies remain needed to compare the therapeutic efficacy of hydrogen/oxygen therapy and oxygen alone.

## Electronic supplementary material

Below is the link to the electronic supplementary material.

Supplementary Material 1

## Data Availability

The datasets used and/or analyzed during the current study are available from the corresponding author upon reasonable request.

## Abbreviations

COVID-19: Coronavirus disease 2019
SARS-CoV-2: severe acute respiratory syndrome coronavirus 2
PSM: propensity-score matched
H_2_-O_2_: hydrogen/oxygen
PCR: polymerase chain reaction
SpO_2_: oxygen saturation
HR: Hazard ratios
CIs: confidence intervals
