# Supplementary material for: Effect of hydrogen/oxygen therapy for ordinary COVID-19 patients: a propensity-score matched case-control study
Source: BMC Infect Dis. 2023 Jun 29;23:440. doi: 10.1186/s12879-023-08424-4 (PMC10308743; doi:10.1186/s12879-023-08424-4)

**Additional file**

**Table S1: Baseline clinical characteristics of patients before propensity score matching or 1:1 matched patients**

|  | **Unmatched patients** | | | **1:1 matched patients** | | |
| --- | --- | --- | --- | --- | --- | --- |
| Variables | Hydrogen/oxygen  group (n=42) | Control group (n=138) | *P* vaule | Hydrogen/oxygen  group (n=42) | Control group (n=138) | *P* vaule |
| Age | 53.1 (11.4) | 56.8 (13.9) | 0.114 | 53.6 (11.0) | 53.43 (13.4) | 0.970 |
| Gender (male/female)-n (%) | 14 (33.3)/28 (66.7) | 64 (46.4)/74 (53.6) | 0.135 | 13 (39.4)/20 (60.6) | 12 (36.4)/21 (63.6) | 0.800 |
| Comorbidities | 0.7 (1.0) | 1.1 (1.4) | 0.092 | - | - | - |
| Days from onset to hospitalization (d) | 26.0 (21.0) | 19.2 (14.5) | 0.043 | 23.6 (12.4) | 25.6 (14.9) | 0.518 |
| Clinical symptoms, yes-n (%) |  |  |  |  |  |  |
| Fever | 2 (4.8) | 26 (18.8) | 0.022 | 2 (6.1) | 5 (15.2) | 0.427 |
| Other symptoms | 32 (76.2) | 95(68.8) | 0.360 | 24 (72.7) | 24 (72.7) | >0.999 |
| Vital signs |  |  |  |  |  |  |
| Respiratory rate (bpm) | 20 (2.0) | 20 (2.0) | 0.611 | 20 (2.0) | 20 (1.0) | 0.812 |
| Heart rate (rpm) | 87.5 (12.6) | 86.4 (13.0) | 0.124 | 86.3 (13.4) | 81.7 (15.2) | 0.200 |
| Systolic pressure (mmHg) | 134 (17.0) | 128 (15.0) | 0.076 | 133 (13.0) | 132 (17.0) | 0.804 |
| Diastolic pressure (mmHg) | 84 (9.0) | 80 (11.0) | 0.017 | 84 (9.0) | 83 (9.0) | 0.809 |
| Temperature (℃) | 36.6 (0.4) | 36.8 (0.7) | 0.054 | 36.6 (0.4) | 36.6 (0.6) | 0.534 |
| Laboratory examination |  |  |  |  |  |  |
| WBC (×10^9^/L) | 5.5 (1.4) | 5. 6 (2.3) | 0.483 | 5.4 (1.4) | 5.3 (1.1) | 0.877 |
| Lymphocyte count (×10^9^/L) | 1.7 (0.4) | 2.5 (10.5) | 0.098 | 1.6 (0.4) | 1.6 (0.4) | 0.963 |
| RBC (×10^12^/L) | 4.3 (0.6) | 4.1 (0.8) | 0.247 | 4.3 (0.7) | 4.2 (0.7) | 0.568 |
| Hb (g/L) | 131 (15.0) | 124 (20.0) | 0.150 | 131 (16) | 127 (19) | 0.862 |
| PLT (×10^9^/L) | 225 (67.0) | 227 (83.0) | 0.896 | 222 (72.0) | 240 (78.0) | 0.344 |
| CRP (mg/L) | 1.6 (2.6) | 6.5 (14.8) | 0.731 | 1.7 (2.9) | 1.2 (1.7) | 0.590 |
| ALT (U/L) | 37 (29.0) | 30(27.0) | 0.051 | 37 (28.0) | 31(27.0) | 0.336 |
| AST (U/L) | 27 (13.0) | 26 (19.0) | 0.203 | 27 (13.0) | 24 (12.0) | 0.422 |
| BUN (mmol/L) | 4.3 (1.2) | 5.5 (3.4) | 0.003 | 4.3 (1.2) | 4.4 (1.3) | 0.646 |
| Scr (μmol/L) | 59.9 (15.0) | 76.5(97.6) | 0.073 | 60.5 (16.3) | 65.1 (14.2) | 0.229 |
| LDH (U/L) | 185 (45.0) | 208 (62.0) | 0.030 | 188 (49.0) | 185 (51.0) | 0.925 |
| Antiviral therapy-n (%) | 9 (21.4) | 70 (50.7) | <0.001 | 9 (27.3) | 9 (27.3) | >0.999 |

Data were expressed as mean (standard deviation) or n (%).

**Figure S1.** Sensitivity analysis of the length of hospitalization.

Panel A showed the Kaplan-Meier curves of the length of hospitalization for hydrogen/oxygen therapy and oxygen therapy in unmatched patients with ordinary COVID-19; Panel B showed the Kaplan-Meier curves of the length of hospitalization for hydrogen/oxygen therapy and oxygen therapy in 1:1 matched patients with ordinary COVID-19; *p* value <0.05 indicates statistical significance. The sensitivity analyses supported the results of primary analysis.


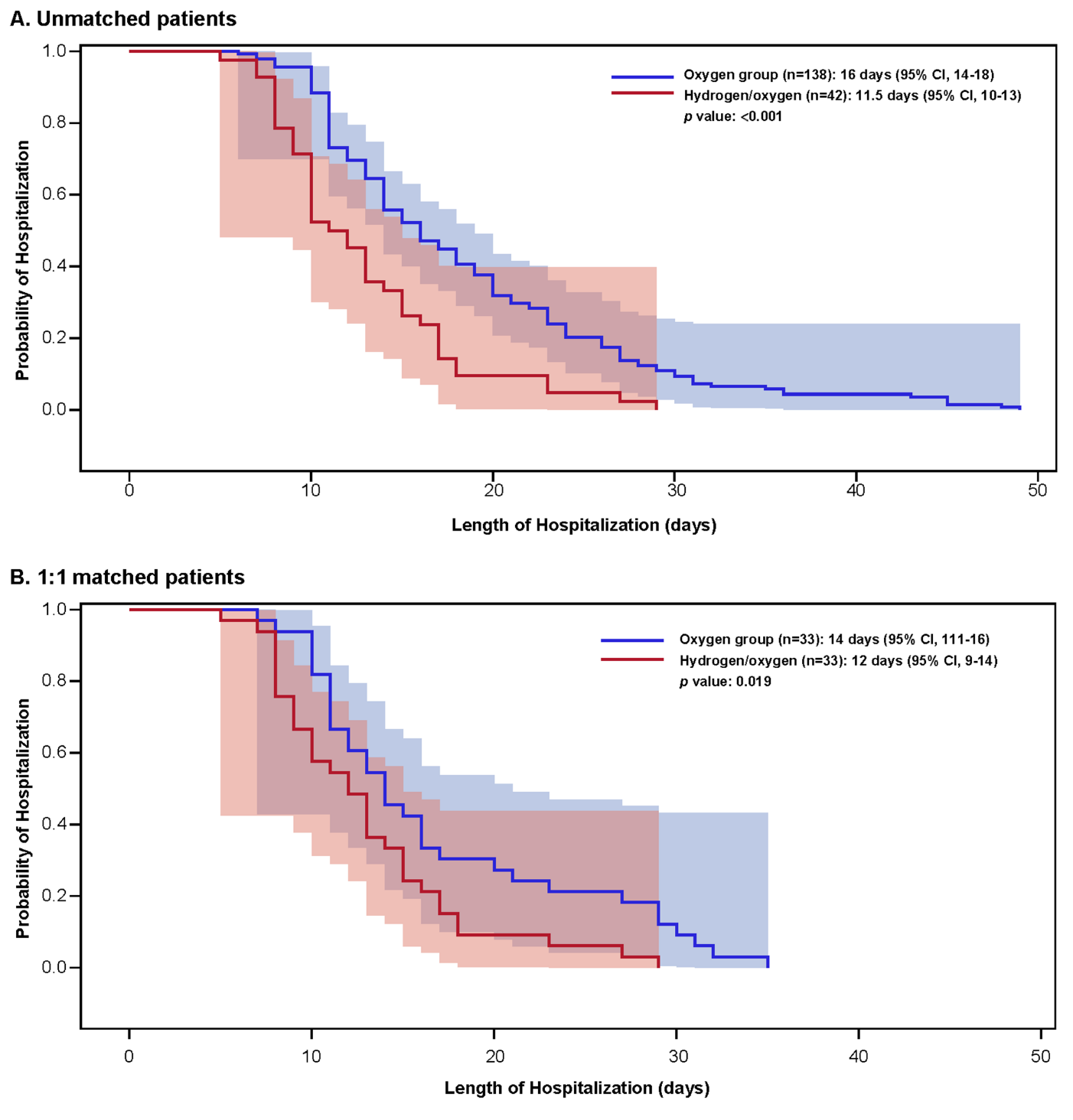

Supplement: Supplementary file 1 — Supplementary Material 1 [file 12879_2023_8424_MOESM1_ESM.docx]
